# Stem Cells Within Three-Dimensional-Printed Scaffolds Facilitate Airway Mucosa and Bone Regeneration and Reconstruction of Maxillary Defects in Rabbits

**DOI:** 10.3390/medicina60122111

**Published:** 2024-12-23

**Authors:** Mi Hyun Lim, Jung Ho Jeon, Sun Hwa Park, Byeong Gon Yun, Seok-Won Kim, Dong-Woo Cho, Jeong Hak Lee, Do Hyun Kim, Sung Won Kim

**Affiliations:** 1 Department of Otolaryngology-Head and Neck Surgery, Seoul St. Mary’s Hospital, College of Medicine, The Catholic University of Korea, Seoul 06591, Republic of Korea; mihyun83@gmail.com (M.H.L.); qscw2002@naver.com (J.H.J.); pooh0523@nate.com (S.H.P.); 2 C&SR Inc., Uiwang 16006, Republic of Korea; bgyun@cnsr.co.kr; 3 Department of Mechanical Engineering, Pohang University of Science and Technology (POSTECH), Pohang 37673, Republic of Korea; tjrjs0531@gmail.com (S.-W.K.); dwcho@postech.ac.kr (D.-W.C.); 4 Yullin St. Mary’s ENT Clinic, Seoul 07639, Republic of Korea; jeonghak70@hanmail.net

**Keywords:** maxillary regeneration, cell transplantation, hNTSCs, mucosal epithelial differentiation, osteogenic differentiation

## Abstract

*Background and Objectives*: Current craniofacial reconstruction surgical methods have limitations because they involve facial deformation. The craniofacial region includes many areas where the mucosa, exposed to air, is closely adjacent to bone, with the maxilla being a prominent example of this structure. Therefore, this study explored whether human neural-crest-derived stem cells (hNTSCs) aid bone and airway mucosal regeneration during craniofacial reconstruction using a rabbit model. *Materials and Methods*: hNTSCs were induced to differentiate into either mucosal epithelial or osteogenic cells in vitro. hNTSCs were seeded into polycaprolactone scaffold (three-dimensionally printed) that were implanted into rabbits with maxillary defects. Four weeks later, tissue regeneration was analyzed via histological evaluation and immunofluorescence staining. *Results*: In vitro, hNTSCs differentiated into both mucosal epithelial and osteogenic cells. hNTSC differentiation into respiratory epithelial cells was confirmed by Alcian Blue staining, cilia in SEM, and increased expression levels of FOXJ1 and E-cadherin through quantitative RT-PCR. hNTSC differentiation into bone was confirmed by Alizarin Red staining, increased mRNA expression levels of BMP2 (6.1-fold) and RUNX2 (2.3-fold) in the hNTSC group compared to the control. Four weeks post-transplantation, the rabbit maxilla was harvested, and H&E, SEM, and immunohistofluorescence staining were performed. H&E staining and SEM showed that new tissue and cilia around the maxillary defect were more prominent in the hNTSC group. Also, the hNTSCs group showed positive immunohistofluorescence staining for acetylated α-tubulin and cytokerin-5 compared to the control group. *Conclusions*: hNTSCs combined with PCL scaffold enhanced the regeneration of mucosal tissue and bone in vitro and promoted mucosal tissue regeneration in the in vivo rabbit model.

## 1. Introduction

Craniofacial defects can be caused by trauma or tumor resection or may be congenital anomalies. Bone and/or soft tissue may be lacking and wounds do not heal. Treatment is difficult because good esthetic results are essential; this is challenging when many different tissue types are in close proximity [1]. The current surgical options include grafts or local tissue rearrangements to repair defects using nearby healthy tissue, as well as microsurgical transfer of tissue [2,3]. However, it is not easy to identify appropriate donor tissues. In addition, donor site morbidity may be of concern.

Three-dimensional (3D)-printed materials may aid bone tissue reconstruction and regeneration [4]. The goal is to fill the defect with structured biomaterial. To date, bone regeneration has received the most attention [5,6,7,8,9]. Also, there has been a report on improving bone regeneration efficiency by prevascularizing scaffolds [10]. However, the craniofacial region includes both skin- and mucosa-covered spaces such as the nasal and oral cavities, the paranasal sinus, the soft palate, and the pharynx. The integrity of a 3D-printed scaffold may be lost on exposure to mucosal secretions such as saliva or nasal materials. Rapid mucosal regeneration is important; it prevents granulated tissue formation and fibrosis, removes foreign substances, and humidifies inhaled air [11,12].

With the advancement of tailored tissue engineering technology, stem cells are being used in a variety of ways, including accelerating healing and reducing scarring in the field of facial reconstruction [13]. During the regenerative process, signaling molecules initiate cellular proliferation to restore the tissue defect and subsequently promote cellular differentiation. These processes are facilitated by growth factors secreted by stem cells.

We previously explored whether human neural-crest-derived stem cells (hNTSCs) harvested from the inferior turbinate could be used to regenerate the airway mucosal epithelium and bone [14,15,16,17]. Through previous research, we found that hNTSCs could be differentiated into bone matrix in vivo [16]. Also, we were able to establish the pore size and porosity conditions for proper engraftment of hNTSCs on a polycaprolactone scaffold using a three-dimensional printing technique [17]. Based on this knowledge, we applied hNTSCs in two distinct forms—airway mucosal epithelial sheets and osteogenically differentiated hNTSCs—to a 3D-printed scaffold and evaluated their effectiveness in reconstructing and regenerating maxillary craniofacial defects in a rabbit model. This approach represents a novel strategy that combines tissue engineering and cell therapy to simultaneously regenerate bone and mucosa, both of which are essential for craniofacial defect repair. It offers a novel solution that distinguishes itself from previous studies on the differentiation potential of hNTSCs.

## 2. Materials and Methods

### 2.1. hNTSC Isolation and Expansion

hNTSCs were obtained following a method reported previously [15], and the protocol was reviewed and approved by the Institutional Review Board of St. Mary’s Hospital, the Catholic University of Korea (approval no. KC08TISS0341). Inferior turbinate tissues were collected from 5 patients undergoing septoplasty. The tissues were rinsed with phosphate-buffered saline (PBS) containing gentamicin and other antibiotic/antimycotic agents, chopped into small pieces (~1 mm^3^), placed in culture dishes, and covered with sterile glass slides. Alpha-minimum essential medium (α-MEM, WelGENE, Daegu, Republic of Korea) containing 1% (*w*/*v*) penicillin/streptomycin (Invitrogen, Carlsbad, CA, USA) and 10% (*v*/*v*) fetal bovine serum (FBS, WelGENE) was added to each plate. The tissues were incubated at 37 °C in a humidified atmosphere with 5% (*v*/*v*) CO_2_. After 3 weeks, non-attached cells were removed from the culture medium, and hNTSCs attached to the plate surfaces were harvested using 0.25% (*w*/*v*) trypsin containing 1 mM EDTA. hNTSCs from five separate culture batches were combined, expanded to passage three, and subsequently utilized for the in vitro study.

### 2.2. Differentiation of hNTSCs at the Air–Liquid Interface (ALI)

ALI cultures were established using the B-ALI^TM^ air–liquid interface medium following the instructions of the manufacturer (Lonza Ltd., Basel, Switzerland). hNTSCs were trypsinized and seeded at 5 × 10^4^ cells/insert onto transwell inserts (0.4 µm pore size membrane; Corning Inc., New York, NY, USA) pre-treated with collagen type I (GenDepot, Barker, TX, USA). Initially, B-ALI^TM^ growth medium (Lonza Ltd.) was added to both the apical and basal chambers. Three days later, the medium was removed from both chambers, and 500 µL B-ALI^TM^ differentiation medium was added exclusively to the basal chamber. The medium was replaced every second day during the 45-day culture period.

### 2.3. Fabrication of an Artificial Maxillary Graft

Polycaprolactone (PCL; molecular weight 43,000–50,000; Polysciences Inc., Warrington, PA, USA) was used to fabricate the 3D constructs, as previously described [17]. Conditions for pore size (300 μm) and PCL thickness (250 μm) that promote cell engraftment on PCL, as well as oxygen plasma treatment at 50 kHz and 100 W for 120 min to enhance the hydrophilicity of the PCL surface, were conducted as described in prior research [18]. PCL granules were loaded into a 10 mL stainless steel syringe, melted at 80 °C, and extruded through a 250 µm diameter nozzle under pneumatic pressure of approximately 650 kPa with a feed rate set at 400 mm/min [19]. The constructs used for in vitro and in vivo experiments had dimensions of 15 mm × 5 mm × 1 mm.

### 2.4. Preparation of hNTSCs Seeded onto Artificial Maxillary Graft

First, prior to in vivo implantation, spinner flasks were used to seed hNTSCs onto artificial maxillary grafts. Each flask contained a base for a graft. The collagen-coated structure was placed on the base, and 2 × 10^6^ cells were added to the flask, which then was transferred to the plate of a magnetic stirrer (Corning) operating at 60 rpm. Incubation proceeded for 3 days at 37 °C in a humidified atmosphere under 5% (*v*/*v*) CO_2_. Then, the cell-seeded maxillary grafts were transferred to non-coated dishes, stabilized in α-MEM medium for 2 days, and stained for F-actin to confirm the cell type. After pre-induction for a further 3 days with osteogenic medium (Gibco BRL, Grand Island, NY, USA), hNTSC sheets were attached to one side of each artificial maxillary graft, and the grafts were transplanted.

Next, hNTSCs sheets were prepared using temperature-responsive culture dishes (35 mm in diameter; Nunc UpCell Surface, Thermo Scientific, Kanagawa, Japan) [16]. hNTSCs were seeded onto the dishes at a density of 1 × 10^5^ cells per well and maintained for 5 days, with the medium being refreshed every other day. Once confluent layer had formed, the hNTSC sheets were detached by lowering the temperature to room temperature for 40 min. Continuous sheets were placed on sterile paper membranes and then attached to artificial maxillary grafts in which osteogenic differentiation had been induced.

### 2.5. RNA Extraction and Quantitative Real-Time PCR

Total RNA was extracted from cultured cells using Trizol reagent (Invitrogen, Carlsbad, CA, USA). For each sample, 1 µg of total RNA was used to generate cDNA with the iScript^TM^ cDNA synthesis kit (Bio-rad, Hercules, CA, USA). Quantitative real-time PCR was used to measure the expression levels of mRNAs encoding genes characteristic of mucosal epithelial and osteogenic differentiation. All reactions proceeded in a CFX96^TM^ Real-Time PCR Detection System (Bio-rad). Each 25 µL reaction mixture contained 2 µL diluted cDNA template, 10 pmol each primer, and 12.5 µL iQ^TM^ SYBR Green Supermix (Bio-rad). The primer sequences are listed in Table 1.

### 2.6. Alcian Blue Staining

Mucin was stained with Alcian Blue. Membranes in the apical chambers were stained with 1% (*w*/*v*) Alcian Blue solution (Sigma-Aldrich, St. Louis, MO, USA) in 3% (*v*/*v*) acetic acid (pH 2.5) for 1 min at room temperature. The dye solution was removed; rinsing with Dulbecco’s phosphate-buffered saline (D-PBS, WelGENE) eliminated nonspecific staining. Photomicrographs were taken using the moticam of a Motic 2000 microscope (Motic, Wetzlar, Germany)

### 2.7. Mineralization Assay

To explore osteogenic differentiation, hNTSC-seeded artificial maxillary grafts were cultured in osteogenic differentiation medium (Gibco BRL) that was changed three times a week. Calcium deposition was detected via Alizarin Red S staining. After 21 days of culture, the grafts were immersed in 4% (*v*/*v*) paraformaldehyde for fixation, followed by rinsing with distilled water. The cells were subsequently stained with 2% (*w*/*v*) Alizarin Red solution (Sigma-Aldrich) for 10 min at room temperature. To quantify mineralization, the stained deposits were dissolved in 10% (*w*/*v*) cetylpyridinium chloride (CPC; Sigma), and the optical density at 550 nm was measured using an ELISA plate reader.

### 2.8. Maxillary Defect Model and Artificial Maxillary Graft Implantation

Four male New Zealand rabbits (OrientBio, Sungnam, Republic of Korea) weighing 2.5–3.5 kg were used. All animal experiments adhered to our institutional guidelines and were approved by the Animal Care and Use Committee of the Catholic University of Korea. Because this study was conducted as a pilot study design, sample size calculation was not performed. Two rabbits received grafts without cells (AMG), and two received grafts with hNTSCs (A-hNTSCs). Rabbits were put under anesthesia through an intramuscular injection of xylazine hydrochloride (6 mg/kg) and ketamine hydrochloride (30 mg/kg). The nasal dorsum was shaved and disinfected with iodine, and a midline incision was created along the dorsum. Then, skin and periosteal flaps were laterally elevated to expose the anterior wall of the maxillary sinus. A maxillary defect of the same size as the artificial maxillary graft, measuring 15 mm × 5 mm × 1 mm, was created. The subcutaneous tissue and skin were closed using absorbable 4–0 vicryl sutures (Ethicon, Somerville, MA, USA). Once the rabbits had recovered from anesthesia, they were permitted unrestricted movement in their cages. Four weeks after implantation, the maxillae were harvested, fixed in 4% (*v*/*v*) paraformaldehyde, and embedded in paraffin.

### 2.9. Histological Analysis and Immunohistofluorescence Staining

Paraffin-embedded sections were initially deparaffinized using xylene and rehydrated through a series of ethanol baths, then stained with hematoxylin and eosin (H&E; Vector Laboratories, Inc., Burlingame, CA, USA) for general morphological evaluation. Before immunofluorescence staining, xylene and ethanol were used to deparaffinize the tissues, followed by rinsing with PBS and immersion in 10 mM citrate buffer (Sigma-Aldrich) at 95–100 °C for 20 min (for antibody retrieval). Then, the sections were incubated overnight at 4 °C with primary antibodies against human acetylated tubulin (1:200; Santa Cruz, Dallas, TX, USA) and cytokeratin-5 (1:200; Abcam, Cambridge, UK) and for 1 h at room temperature with secondary antibodies: Alexa Fluor 488 (green)- or 546 (red)-conjugated goat anti-rabbit IgG or mouse IgG (1:2000; Thermo Fisher Scientific, Waltham, MA, USA). All samples were then counterstained with 4′,6-diamidino-2-phenylindole (DAPI; Sigma-Aldrich), and fluorescence images were acquired using a confocal microscope (LSM800 w/Airyscam; Carl Zeiss Meditec AG, Jena, Germany).

### 2.10. Scanning Electron Microscopy (SEM)

We observed regenerated cilia in both in vitro and in vivo models. To prepare for scanning electron microscopy (SEM) analysis, samples were fixed in 2.5% glutaraldehyde at 4 °C, followed by washing and dehydration through a graded ethanol series (50% to 100%). Then, the samples were treated serially with 25%, 50%, and 75% isoamyl acetate in ethanol and, finally, with 100 % isoamyl acetate. The samples were then dried by critical point drying (HDP-2; Hitachi, Tokyo, Japan), coated with platinum, and observed using SEM (S4700; Hitachi, Tokyo, Japan) at Eulji University.

### 2.11. Statistical Analysis

All data are means ± SDs of the results of at least three independent experiments. Statistical differences were identified using the *t*-test. A *p*-value < 0.05 was considered significant. Prism ver. 5.0 software (GraphPad Software, San Diego, CA, USA) was used to perform all statistical analyses.

## 3. Results

### 3.1. Differentiation of Respiratory Epithelial Cells from hNTSCs

**Figure 1:**
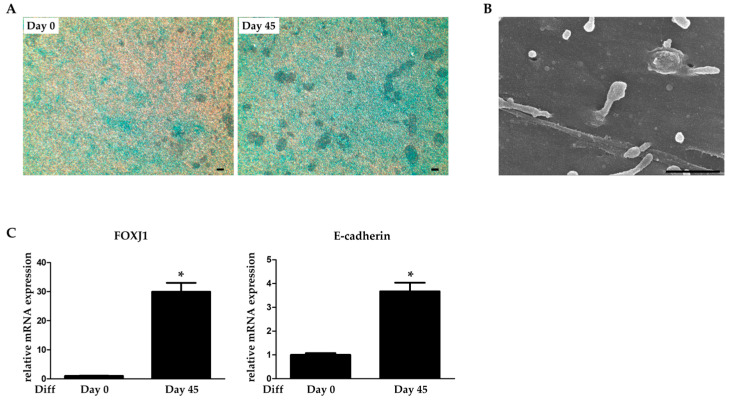
Differentiation of hNTSCs into epithelial cells. (**A**) Alcian Blue staining revealing mucus production. Scale bars: 100 µm (**B**) SEM of cilia. Scale bars: 1.0 µm. (**C**) Real-time PCR data derived on days 0 and 45 after epithelial differentiation were induced. Bars: Relative expression levels (±SDs). * *p* < 0.05.

Passage 3 hNTSCs were seeded into transwell inserts at 4 × 10^5^ cells/well. Following 3 days of culture, once the cells were confluent, the medium was replaced with an ALI differentiation medium. After 45 days of differentiation into airway epithelial cells, Alcian Blue staining revealed mucus on the cell surfaces (Figure 1A); SEM confirmed that cilia were present (Figure 1B). Quantitative RT-PCR analysis demonstrated, as expected, that the expression levels of FOXJ1 (a cilium-related gene) and E-cadherin (a tight junction gene) were higher in hNTSCs cultured under ALI conditions for 45 days than in day 0 cells (Figure 1C). Thus, hNTSCs differentiated in vitro into respiratory epithelial cells that produced both mucus and cilia.

### 3.2. Cell Distribution and Osteogenic Differentiation of hNTSCs on an Artificial Maxillary Graft In Vitro

**Figure 2:**
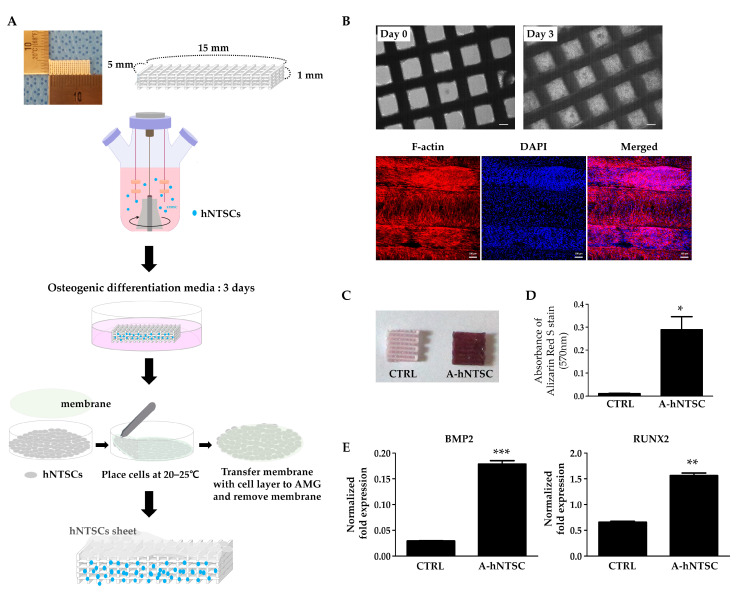
Cell seeding into artificial maxillary grafts and pre-implantation culture. (**A**) hNTSC seeding into AMG in a spinner flask. hNTSC sheets covered the AMG after 3 days of culture in osteogenic induction medium before implantation of AMG-hNTSCs into rabbits. (**B**) An optical microscopic image of AMG seeded with hNTSCs after 3 days of culture (upper panel) and a confocal microscope image (with z-stack projections) after staining for F-actin (red). The nuclei were stained with DAPI (blue). Scale bars: 200 and 100 µm, respectively. (**C**) Images of CTRL (non-induced A-hNTSCs) and A-hNTSCs stained with Alizarin Red S, a dye used to detect calcium deposition, after 21 days of incubation in osteogenic differentiation medium. (**D**) The mineralization was quantified by extraction of Alizarin Red S dye using the CPC extraction method, and absorbance was measured at 570 nm. * *p* < 0.05 compared with CTRL group. (**E**) The expression levels of BMP2 and RUNX2 after 21 days of incubation in osteogenic differentiation medium as revealed by real-time PCR. Bars: Relative expression levels (±SDs). ** *p* < 0.01 and *** *p* < 0.001.

The preparation of an artificial maxillary graft for transplantation into the rabbit maxilla is shown in Figure 2A. Using a spinner flask, cell-seeded grafts were transferred to non-coated plates, stabilized, and cultured for 3 days; then, the cell distribution was confirmed. Immediately after removing them from the spinner flask, only a few cells were apparent between the grafts; however, following 3 days of incubation, the cells were well-distributed throughout the grafts. To visualize the actin structure, the distribution of F-actin, a major cytoskeletal component, was investigated via staining. hNTSCs were evenly distributed within the graft space and on the graft surface (Figure 2B).

To investigate the osteogenic differentiation potential of hNTSCs seeded on the artificial maxillary graft, osteogenesis was induced in vitro for 21 days. Alizarin Red S staining showed that A-hNTSCs cultured in an osteogenic induction medium had more detectable calcium mineral nodules compared to A-hNTSCs cultured in a non-induction medium (CTRL) (Figure 2C). A quantitative assessment of mineralization was performed by analyzing the optical density (OD) values of the dissolved stains using CPC (Figure 2D). As shown in the graph in Figure 2D, higher values were obtained in the osteogenic AMG-hNTSC group (*p* < 0.05). Additionally, on day 21 of induction, the mRNA expression levels of BMP2 and RUNX2 were increased approximately 6.1-fold and 2.3-fold, respectively, in the induced AMG-hNTSCs compared to the non-induced AMG-hNTSCs (CTRL), indicating that the osteogenic differentiation potential of hNTSCs is maintained under in vitro 3D environmental conditions (Figure 2E).

### 3.3. Orthotopic Implantation of AMG-hNTSCs into Maxillary Defects

**Figure 3:**
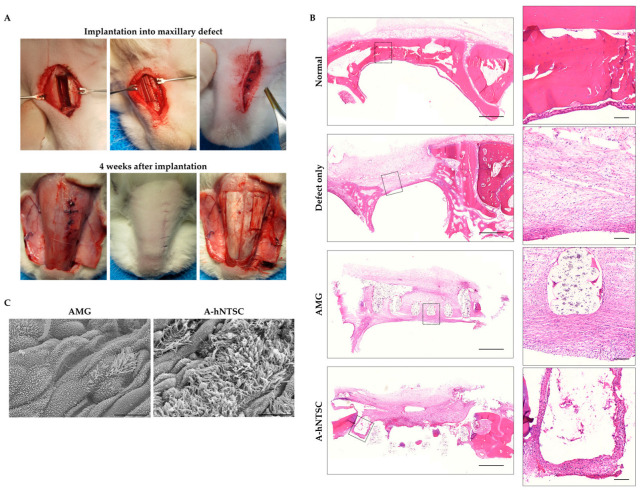
Surgical and sacrifice procedures and evaluation of ciliary regeneration. (**A**) Images taken during surgery (**top**) and sacrifice (**bottom**). (**B**) H&E staining of paraffin-embedded sections (scale bars: 1000 µm and 100 µm, respectively), and (**C**) SEM images obtained at 4 weeks after implantation of AMG or A-hNTSCs. Scale bars: 10 µm.

**Figure 4:**
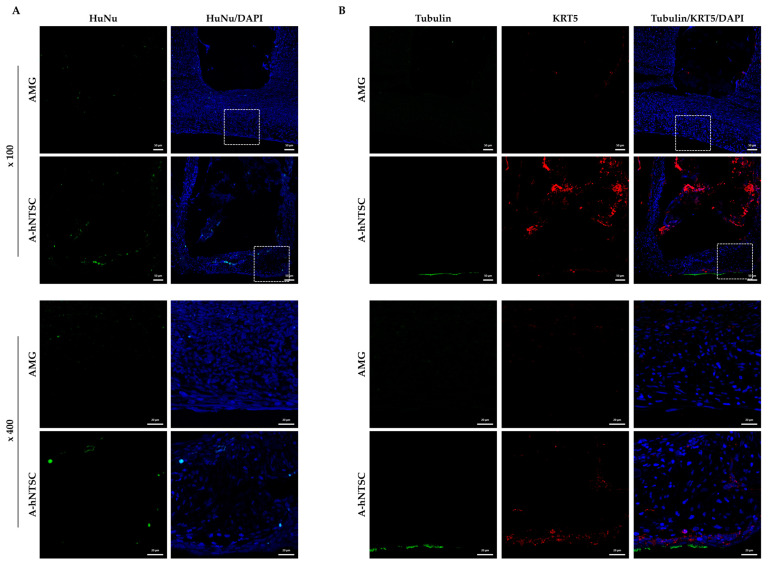
Immunohistofluorescence staining of hNTSCs in maxillary defects after implantation of AMG or A-hNTSCs. Confocal microscopy images (z-stack projections) of AMG and A-hNTSCs after staining of paraffin-embedded sections (a) with an antibody against (**A**) HuNu and (**B**) double-staining with antibodies against acetylated α-tubulin (green) and cytokeratin-5 (red) at 4 weeks. The nuclei were labeled with DAPI (blue). Scale bars: (**A**,**B**): upper panels 50 µm; lower panels 20 µm.

AMG alone or A-hNTSCs were orthotopically implanted into the maxillary defects of rabbits (Figure 3A). Four weeks after transplantation of AMG or A-hNTSCs, both flaps were elevated to observe the grafted and non-grafted areas, verifying the healing with new mucosa at the surgical sites. H&E staining revealed new tissue around the maxillary defects in all rabbit models; however, cilia were observed only in the model implanted with A-hNTSCs (Figure 3B). Bone formation was not detected. SEM revealed that all defects were covered with ciliated epithelium. However, apical surface buds, characteristic of early epithelial ciliary development, were more prominent in the AMG group, whereas the A-hNTSCs group exhibited more and longer cilia (Figure 3C). In addition, implanted human cells were apparent around the A-hNTSCs; these cells were immunopositive to acetylated α-tubulin and cytokerin-5 (Figure 4). Although further work is needed to determine whether hNTSCs directly differentiate into mucosal epithelial cells in vivo, the differences in ciliary morphology and immunopositivity between the two groups imply that hNTSCs aid mucosal epithelial regeneration.

## 4. Discussion

We explored whether hNTSC cell sheets facilitate maxillary defect repair. Regeneration using adult stem cells eliminates the need for ethical debates on the clinical application of embryonic stem cells [23]. The potential clinical applications of stem cells extracted from adult bone marrow and other adult tissues have been extensively studied for decades [23,24,25]. However, collecting them entails painful surgery, and the yields are low.

Surgical procedures on the turbinates are among the most frequently performed operations worldwide [26]; inferior turbinate tissues are usually discarded (they are “surgical waste”). hNTSCs can be readily isolated from such tissues. In addition, hNTSCs are cells of the neural-crest-derived HOX-negative facial skeleton and exhibit a greater self-renewal capacity and environmental plasticity than fully differentiated adult cells [27,28]. hNTSCs can be obtained at a higher yield than bone marrow- and adipose-tissue-associated mesenchymal stem cells (MSCs); they have robust differentiation and proliferation capacities regardless of donor age or passage number [14,15]. Thus, these cells may usefully aid epithelial regeneration [17]. Here, we explored whether hNTSCs exhibiting both osteogenic and epithelial regeneration capacities might be useful for treating craniofacial defects by seeding these cells onto the PCL-based scaffold [29]. PCL is an FDA-approved, biocompatible polymer that safely degrades in the body over 3–4 years. It is highly suitable for 3D printing, allowing for the creation of patient-specific implants. PCL melts at 60 °C and can be extruded layer by layer without toxic solvents, making it ideal for custom anatomical designs [30,31]. Tissue engineering requires large numbers of cells; the chosen cells must proliferate readily. Low-level cell proliferation often limits potential therapeutic applications. We found that hNTSCs attached to an artificial construct and proliferated well when cultured in spinner flasks (Figure 2). Remarkably, stem cells can differentiate into diverse tissues in vitro, implying that transplanted stem cells could repair damaged tissues by generating new cells at the site of injury [32,33].

Several studies have shown that MSC transplantation supports epithelial regeneration, stimulates osteogenesis, and facilitates the repair of bone defects [34,35]. Given the functional relationship between the epithelial and bone-forming differentiation of MSCs in vitro and the role played by MSCs in in vivo regeneration, we studied the distinct differentiation of such cells in vitro. hNTSCs exhibited marked ciliogenesis and high-level expression of epithelial-cell-related genes, including FOXJ1 and E-cadherin; they differentiated into functional epithelial cells (Figure 1). We found that the mucosa of animals implanted in vivo with hNTSC-seeded artificial maxillary grafts expressed more cilia compared to those of controls, consistent with the in vitro results indicating that hNTSCs exhibited epithelial differentiation (Figure 4). Furthermore, the osteogenic differentiation of hNTSCs seeded into artificial maxillary grafts in vitro was associated with increased mineral deposition and upregulated expression of genes related to bone development, including BMP-2 and RUNX2 (Figure 2). However, in contrast to what was noted in vitro, bone formation was not observed in vivo. Several reports have described bone formation at approximately 4–12 weeks after implants were used to remedy bone defects in rabbits. It may be that the large defects that we created, followed by early sacrifice, explain why bone formation was not observed [36,37]. Future studies should evaluate bone formation at later time points. In this study, we found that artificial maxillary grafts containing hNTSCs enhanced mucosal epithelial regeneration and bone formation both in vitro and in vivo. hNTSCs differentiate well into mucosal tissue because the cells are derived from the mucosa of the human craniofacial region [17]. They can be readily induced to undergo osteogenic differentiation in vitro or in hydrogels in vivo [14,16]. Therefore, we suggest that hNTSCs can be used to repair maxillary defects.

Through this study, we were able to present a meaningful alternative for maxilla reconstruction by using hNTSCs together with a PCL scaffold. However, this study has several limitations. First, with only four animals, the sample size is limited, potentially weakening the statistical significance and reliability of the outcomes. This restricts the generalizability of the findings, highlighting the importance of future studies with larger samples. Second, anatomical and physiological differences between rabbits and humans can impact the applicability of the findings to clinical settings. Specifically, structural variations, healing speeds, and immune responses in the maxilla differ between species, which can limit how directly these results translate to humans. Third, although we observed a 4-week course and rabbits have a very rapid regeneration rate compared to humans, the limited study period is likely insufficient to observe complete regeneration and integration of bone and mucosa over time.

Stem cell therapy and tissue engineering have shown promise in advancing facial reconstruction; however, these approaches currently face a range of challenges that must be overcome to achieve clinical viability. One primary issue lies in the reliable control of stem cell differentiation and integration within host tissues, as inconsistent outcomes can lead to regeneration irregularities or the production of unintended cell types. Additionally, replicating the intricate vascular and neural networks that are necessary for functional facial tissue has proven difficult, often resulting in incomplete or functionally limited tissue repair. Further, concerns about the immunogenicity and compatibility of tissue-engineering scaffolds remain. Despite progress in developing materials intended to reduce immune rejection and inflammation, current scaffolding technologies still struggle to fully support cell growth while integrating smoothly with native tissue. Scalability is also an obstacle; existing methods frequently fail to produce consistent results tailored to the anatomical and functional diversity required in facial reconstruction. Future advances may address these limitations through improved genetic and molecular engineering techniques, allowing more precise control over stem cell behavior and scaffold interactions. In particular, developments in 3D bioprinting hold the potential for producing facial structures that can incorporate complex vascular systems, thereby enhancing functionality and integration. In addition, research focused on the microenvironmental factors that influence stem cell differentiation and integration could lead to improved approaches for stem cell therapy. Moving forward, interdisciplinary collaboration and thorough clinical testing will be essential to establish stem cell and tissue-engineering techniques as viable options in facial reconstruction.

## 5. Conclusions

hNTSCs differentiated into both respiratory epithelial and osteogenic cells. They attached to and proliferated within artificial maxillary grafts, mucus was produced, and cilia were formed in vitro. The cells, when seeded into artificial maxillary grafts, exhibited osteogenic differentiation, as confirmed by both mineral deposition and increased expression of the osteogenic markers BMP2 and RUNX2. In vivo, artificial maxillary grafts seeded with the cells enhanced epithelial regeneration. The cilia were longer and more abundant than those of controls. Together, the findings imply that hNTSCs may facilitate both epithelial and bone regeneration and will find valuable clinical applications.

## Figures and Tables

**Table 1:** Primer sequences used in real-time PCR.

Name	Forward Primer (5′→3′)	Reverse Primer (5′→3′)	Ref.
FOXJ1	GAGCGGCGCTTTCAAGAAG	GGCCTCGGTATTCACCGTC	[20]
E-cadherin	CCCACCACGTACAAGGGTC	CTGGGGTATTGGGGGCATC	[21]
BMP2	TTCCACCATGAAGAATCTTTGGA	CCTGAAGCTCTGCTGAGGTGAT	[22]
RUNX2	TCCTCCCCAAGTAGCTACCT	AGCTTCTGTCTGTGCCTTCT	[22]
GAPDH	AACAGCGACACCCACTCCTC	CATACCAGGAAATGAGCTTGACAA	[22]

## Data Availability

The raw data will be provided by the authors upon request.
